# Supplementary material for: C-terminal calcium binding of α-synuclein modulates synaptic vesicle interaction
Source: Nat Commun. 2018 Feb 19;9:712. doi: 10.1038/s41467-018-03111-4 (PMC5818535; doi:10.1038/s41467-018-03111-4)
Supplement: Supplementary file 1 — Supplementary Information [file 41467_2018_3111_MOESM1_ESM.pdf]

Calcium binding at the C-terminus of α-synuclein modulates synaptic vesicle interaction

Lautenschläger et al.

Supplementary Figures

Supplementary Figure 1

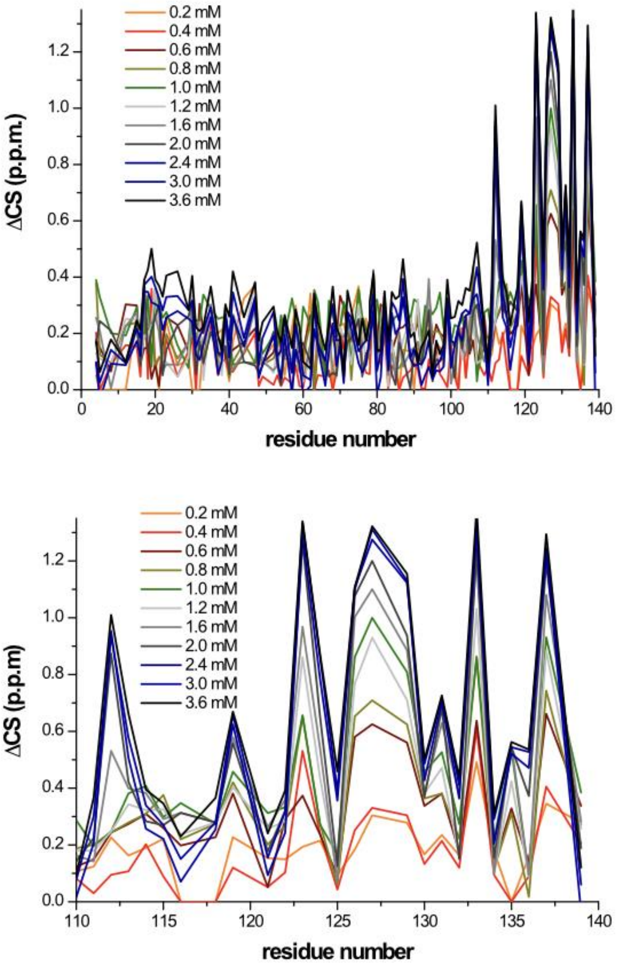

^{1}H-^{15}N HSQC NMR spectra were measured at increasing calcium concentrations. Chemical shift changes for the full protein (upper panel) and zoomed in for amino acids 110 to 140 (lower panel) are plotted against alpha-synuclein residue number. Saturation of signal changes occurred at 3.6 mM calcium.

Supplementary Figure 2

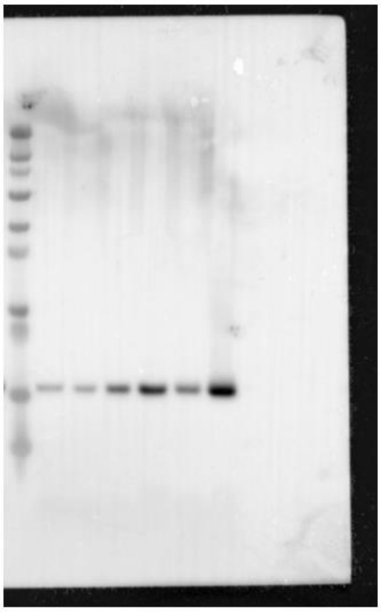

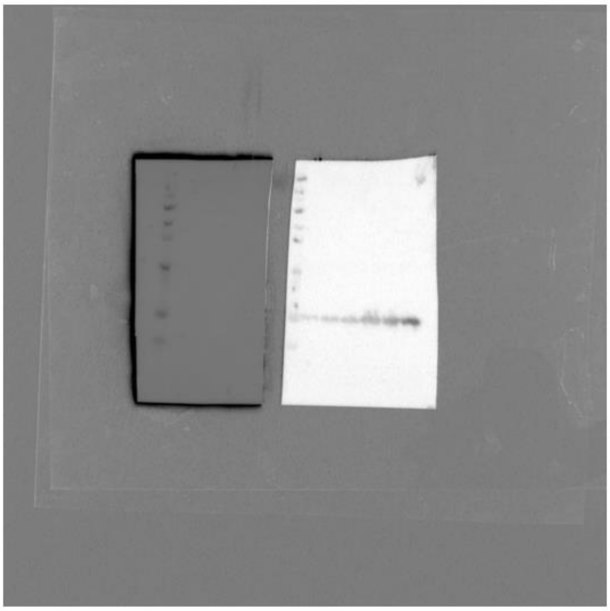

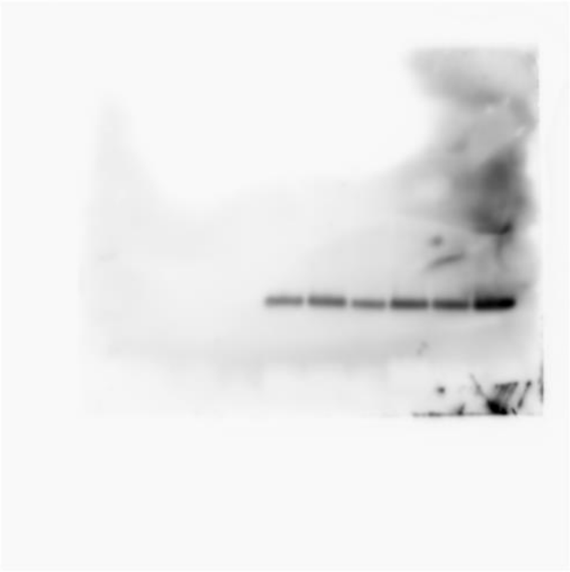

Original Western blot images corresponding to data shown in Figure 1d.

Supplementary Figure 3

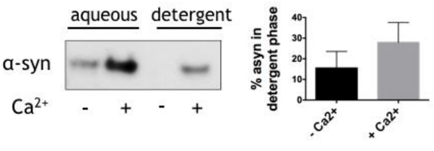

Phase partitioning assay using Triton X-114 probing the hydrophobicity of alpha-synuclein in the absence and presence of calcium. Western blot against alpha-synuclein shows that the amount of alpha-synuclein in the detergent phase increased from 15.51 +/- 8.03 % to 27.96 +/- 9.61 % upon calcium exposure revealed as percentage of alpha-synuclein in the aqueous as well as detergent phase. N = 3 for both groups corresponding to 3 biological repeats. Error bars indicate sem.

Supplementary Figure 4

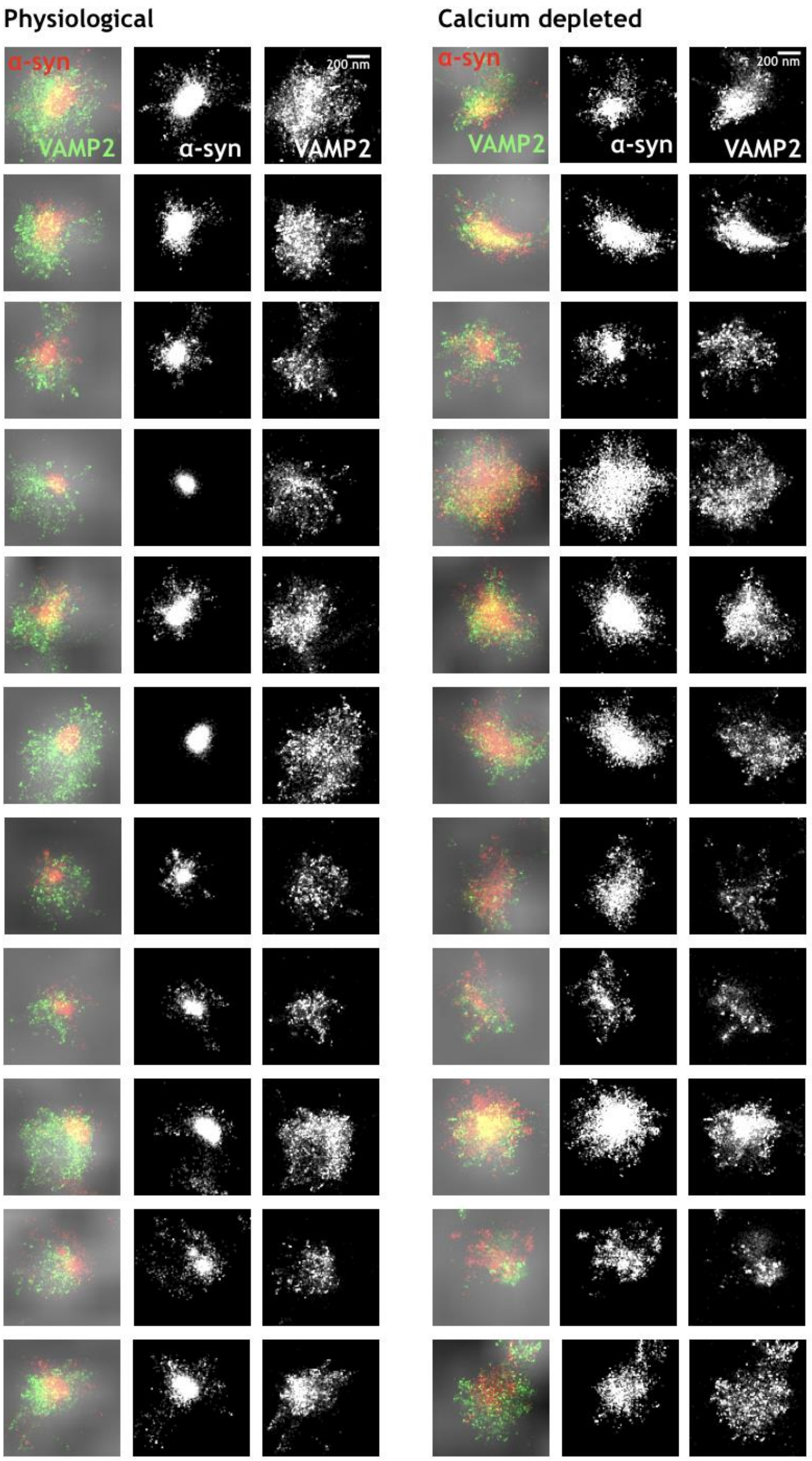

Compilation of individual images of synaptosomes as used for Figure 2d.

Supplementary Figure 5

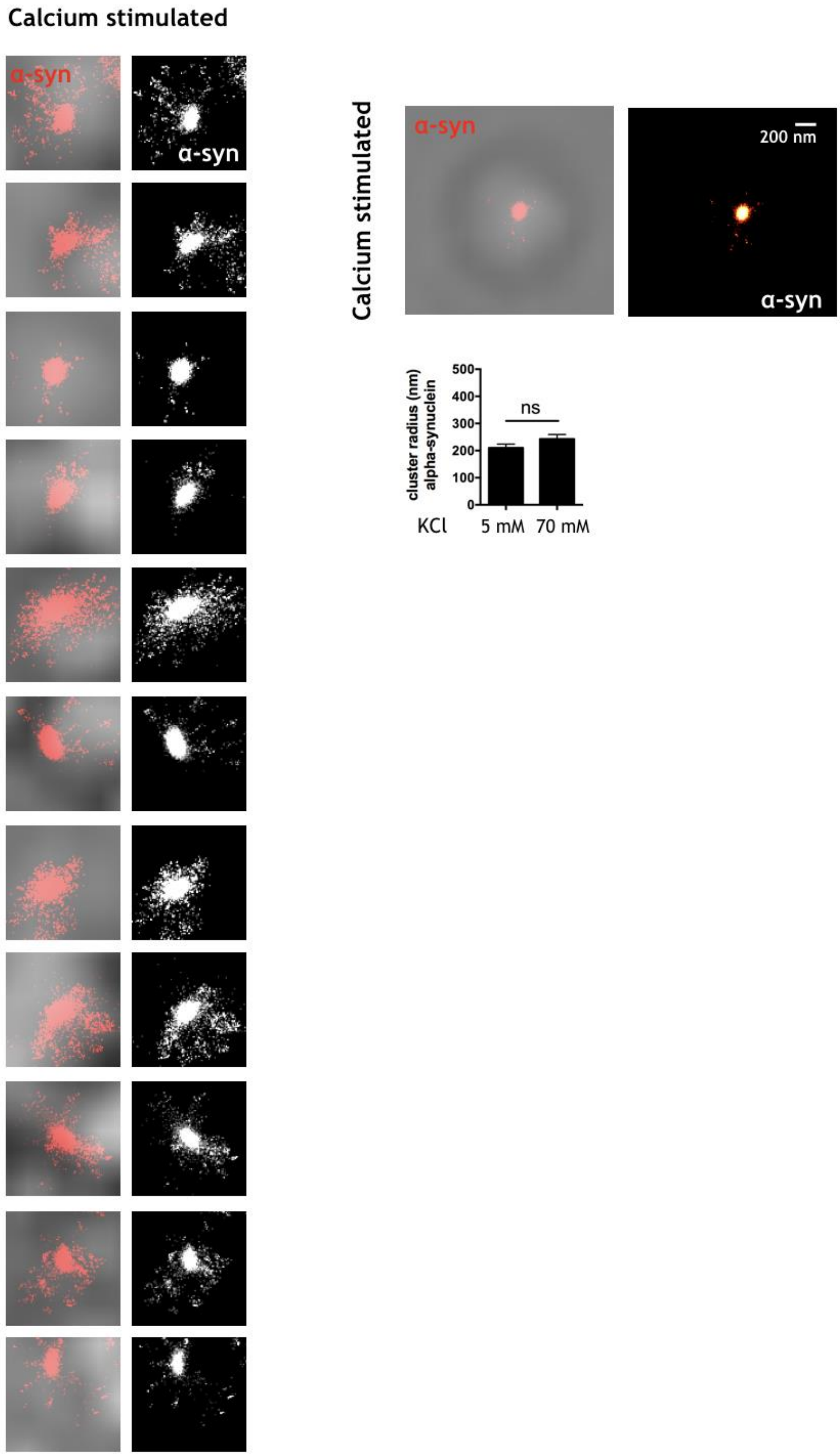

*(a) d*STORM super-resolution imaging of alpha-synuclein in isolated synaptosomes stimulated with extracellular solution at 70 mM KCl. (b) There was no significant difference in alpha-synuclein cluster size between unstimulated versus KCl stimulated synaptosomes (5 mM KCl vs 70 mM KCl; 2.5 mM $CaCl_2$). $^{ns}p = 0.1427$, calculated using two-tailed t-test, graphs indicate mean +/- s.e.m.. N = 22 for 5 mM KCl and n = 20 for 70 mM KCl, where n indicates single synaptosomes, data from 3 biological repeats, d.f. 40.

Supplementary Figure 6

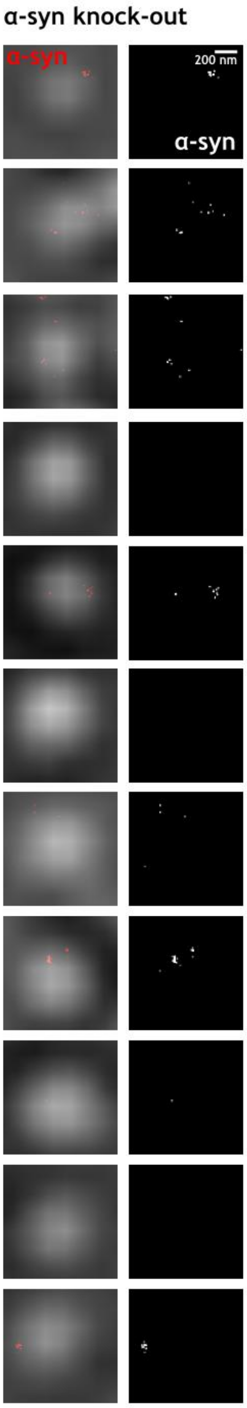

Compilation of individual image of synaptosomes obtained from alpha-synuclein knock-out mice under physiological conditions.

Supplementary Figure 7

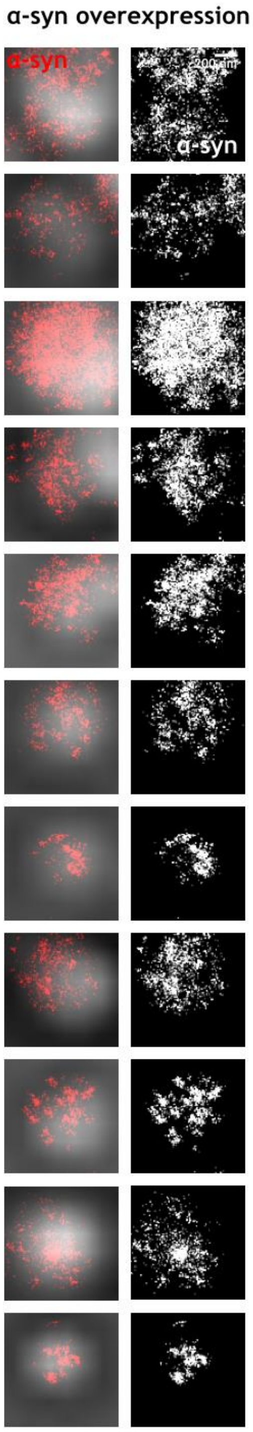

Compilation of individual images of synaptosomes obtained from alpha-synuclein overexpressing mice under physiological conditions.

Supplementary Figure 8

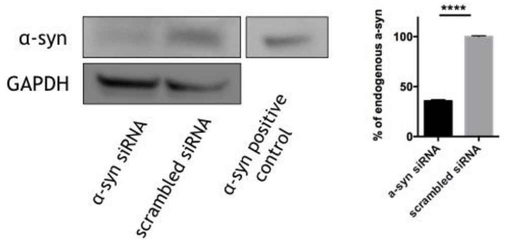

Western blot for alpha-synuclein knock-down in SH-SY5Y cells showing a reduction of alpha-synuclein levels of over 50 %. ***$p < 0.001$, calculated using two-tailed t-test, n = 3 for both groups, d.f. 4, error bars indicate sem.

Supplementary Figure 9

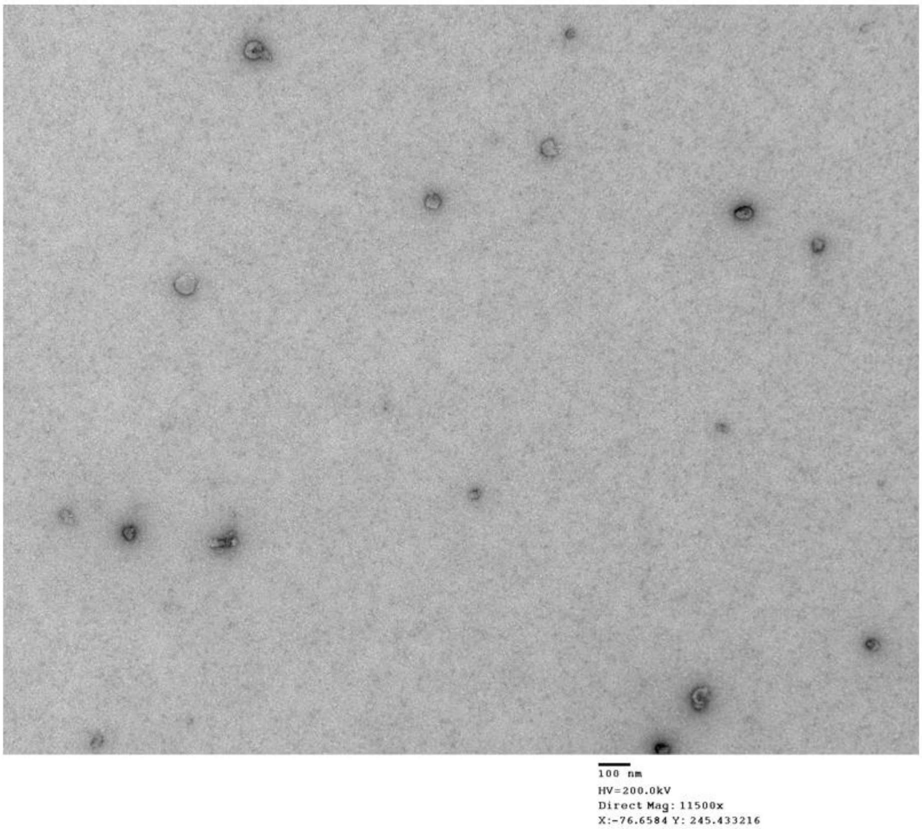

TEM image of alpha-synuclein in the presence of synaptic vesicles at the beginning of the aggregation experiment shown in Figure 5b.

Supplementary Tables

Supplementary Table 1

		α-syn		α-syn + $CaCl_2$	
Species	Calculated mass (Da)	Observed mass (Da)	Error (Da)	Observed mass (Da)	Error (Da)
α-syn	14460.19	14460.38	0.20	14460.24	0.06
α-syn.Ca	14498.25	14497.44	-0.81	14498.10	-0.15
α-syn.2Ca	14536.31	- §	- §	14535.61	-0.70
α-syn.3Ca	14574.37	- §	- §	14573.19	-1.18
α-syn.4Ca	14612.43	- §	- §	14611.18	-1.25
α-syn.5Ca	14650.50	- §	- §	14649.22	-1.28
α-syn.6Ca	14688.56	- §	- §	14687.26	-1.30

Calcium-bound alpha-synuclein species observed by mass spectrometry as shown in Figure 1C. Observed masses were determined by charge deconvolution of the ion envelope for the 9^+ - 19^+ charge states, inclusive. Limit of detection was taken as 1% of base peak (most abundant species) intensity. Alpha-synuclein: calcium complexes were not observed above a 1:1 stoichiometry in the absence of incubation with $CaCl_2$.

Supplementary Methods

Phase partitioning assay

Phase separation of proteins was performed as described by Davletov et al. using Triton X-114, which separates into an aqueous phase and a detergent phase above a temperature of 20 °C [1]. If hydrophobic portions of a protein are exposed, the protein will partition into the detergent phase [2]. For the assay, an aliquot of 55 µL of 10% Triton X-114 (Sigma-Aldrich) was added to 500 µL of buffer A (150 mM NaCl, 2 mM Na_2EDTA, 20 mM Tris, pH 7.4) containing 15 µg alpha-synuclein in the presence or absence of 2.2 mM $CaCl_2$. The final pellet was dissolved in 80 µL NuPAGE® LDS Sample Buffer (Life Technologies, Paisley, UK). Details for Western blot of the phase partitioning assay are the same as found under Lipid pull down assay.

Alpha-synuclein knock-out

Knock-out of alpha-synuclein was performed using the pSilencer™ 4.1-CMV neo Kit (Ambion, Life Technologies). Oligonucleotide templates were designed according to the manufacturer's instructions. Following hairpin siRNA template oligonucleotides were used:

siRNA hairpin 1: 5'- AGAGCAAGTGACAAATGTTCTCAAGAGAAACATTTGTCACTTGCTCTTT-3'

siRNA hairpin 2: 5'- GGAATTCTGGAAGATATGCTTCAAGAGAGCATATCTTCCAGAATTCCTT-3'

siRNA hairpin 3: 5'- TTCTGGAAGATATGCCTGTTTCAAGAGACAGGCATATCTTCCAGAATT-3'

siRNA hairpin 4: 5'- TGAGGCTTATGAAATGCCTTTCAAGAGAGGCATTTCATAAGCCTCATT-3'

The negative control vector, with a scrambled targeting sequence, was supplied as part of the pSilencerTM 4.1-CMV neo kit. SH-SY5Y cells were electroporated using Amaxa Cell Line Nucleofactor Kit V (Lonza, Slough, UK) according to the manufacturer's instructions.

Transgenic animals

Synaptosomes were isolated from SNCA null mice and mice expressing human wild-type alpha-synuclein. SNCA null mice were obtained from the Jackson Laboratory [3], human wild-type alpha-synuclein were crossed with the SNCA mice to create transgenic lines lacking endogenous murine alpha-synuclein [4]. All animal experiments were performed according to guidelines established in the Canadian Guide for the Care and Use of Laboratory Animals.

Code for image analysis of synaptic vesicle STED images

```
% Plots histogram of distance of XY points
% XY points can be generated from Icy spot detection plugin, then
% saved as a csv file.
  %
% Marcus Fantham, mjf74, 2017

function histogramDistancePlotter()
%% SET DISTANCE HERE!!
r = 12.5; % pixels

%% Open file
[file, path] = uigetfile('.csv', 'Choose CSV file of XY coordinates');
if file == 0, return; end
filename = fullfile(path, file);

%% Import data
delimiter = ',';
formatSpec = '% f % f %[^\n\r]';
fileID = fopen(filename,'r');
dataArray = textscan(fileID, formatSpec, 'Delimiter', delimiter,
'ReturnOnError', false);
fclose(fileID);

x = dataArray{:, 1};
y = dataArray{:, 2};

%% Useful variables
N = length(x);
X = [x,y];

%% Find distance
rngSch = rangesearch(X,X,r, 'Distance', 'minkowski', 'NSMethod',
'exhaustive');

%% Make histogram table
histo = zeros(1,N);
for n = 1:N
    histo(n) = length(rngSch{n});
end

%% Plot histogram
histogram(histo);
xlabel(sprintf('Number of neighbours within % .1f pixels', r));
ylabel('Number of spots like this');

%% Print histogram nicely to screen
[N, edges] = histcounts(histo);
bins = (edges(2:end)-0.5)';
disp([bins, N']);
```

Supplementary References

1. Davletov, B., Perisic, O. & Williams, R. L. Calcium-dependent Membrane Penetration Is a Hallmark of the C2 Domain of Cytosolic Phospholipase A2 Whereas the C2A Domain of Synaptotagmin Binds Membranes Electrostatically J. Biol. Chem. **273,** 19093–19096 (1998).

2. Nalefski, E. A. *et al.* Independent Folding and Ligand Specificity of the C2 Calciumdependent Lipid Binding Domain of Cytosolic Independent Folding and Ligand Specificity of the C2 Calcium- dependent Lipid Binding Domain of Cytosolic Phospholipase A2. *J. Biol. Chem.* **273,** 1365–1372 (1998).

3. Abeliovich, A. *et al.* Mice lacking alpha-synuclein display functional deficits in the nigrostriatal dopamine system. *Neuron* **25,** 239–252 (2000).

4. Wislet-Gendebien, S. *et al.* Cytosolic proteins regulate α-synuclein dissociation from presynaptic membranes. *J. Biol. Chem.* **281,** 32148–32155 (2006).
